# Leiomyoma originating from left round ligament presents as symptomatic inguinal hernia

**DOI:** 10.1002/ccr3.4445

**Published:** 2021-07-19

**Authors:** Konstantinos Kypriotis, Nikolaos Kathopoulis, Maria Tsiriva, Dimitrios Zacharakis, Ioannis Chatzipapas, Athanasios Protopapas

**Affiliations:** ^1^ 1st Department of Obstetrics and Gynecology Faculty of Medicine National and Kapodistrian University of Athens “Alexandra” Hospital Athens Greece

**Keywords:** inguinal hernia, laparoscopy, round ligament leiomyoma

## Abstract

Leiomyomas may develop at extra‐uterine locations and pose diagnostic dile mmas. This is a case of a fibroma originating from the left round ligament presenting as a symptomatic inguinal hernia.

## Graphical abstract

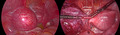

Leiomyomas are common uterine tumors. Occasionally, they may be found at unusual extrauterine locations developing from embryonic remnants or nonuterine pelvic smooth muscle causing problems in the differential diagnosis.^1^, ^2^ We present the rare case of a 26‐year‐old patient with a leiomyoma originating from the left round ligament, independently from the uterus. The patient presented with progressively deteriorating left‐sided discomfort, aggravated during exercise and heavy weight lifting. Pelvic ultrasonography revealed the presence of a solid tumor measuring 7.5 × 6.6 × 5.9 cm. No apparent connection to the uterus was detected, and differential diagnosis included pedunculated or inter‐ligamentous uterine leiomyoma versus ovarian fibroma or thecoma. At laparoscopy, it was found developing retroperitoneally 2 cm from the left side of the uterus, independently of it and the ipsilateral ovary (Figure 1). It was densely adhered to the antero‐lateral pelvic wall, the underlying retroperitoneal tissues, and the rectosigmoid. After careful retroperitoneal dissection, avoiding traumatization of the tumor through direct grasping, the proximal and distal parts of the round ligament were coagulated and cut, resulting in its complete mobilization (Figure 2). The exposed left inguinal hernia (Figure 3) which resulted from chronic tumor compression was repaired with three interrupted nonabsorbable sutures.

**FIGURE 1:**
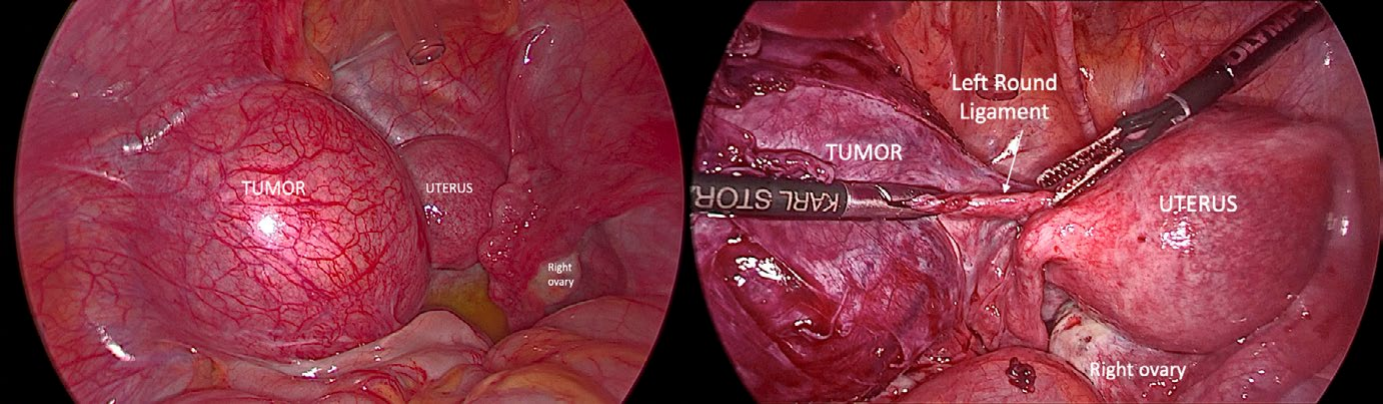
The tumor is developing retroperitoneally 2cm from the left side of the uterus, independently of it and the ipsilateral ovary

**FIGURE 2:**
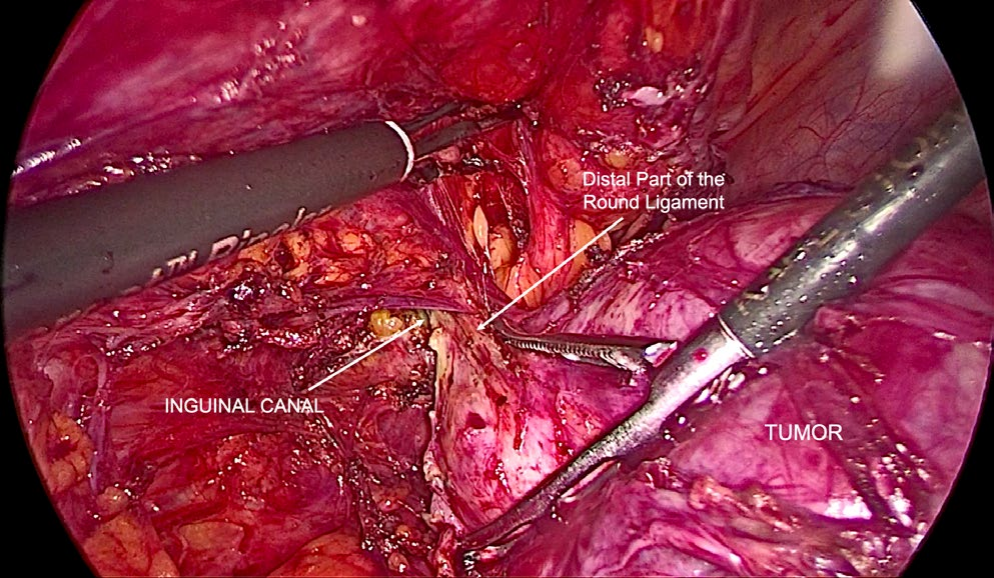
Complete mobilization of the tumor after careful retroperitoneal dissection

**FIGURE 3:**
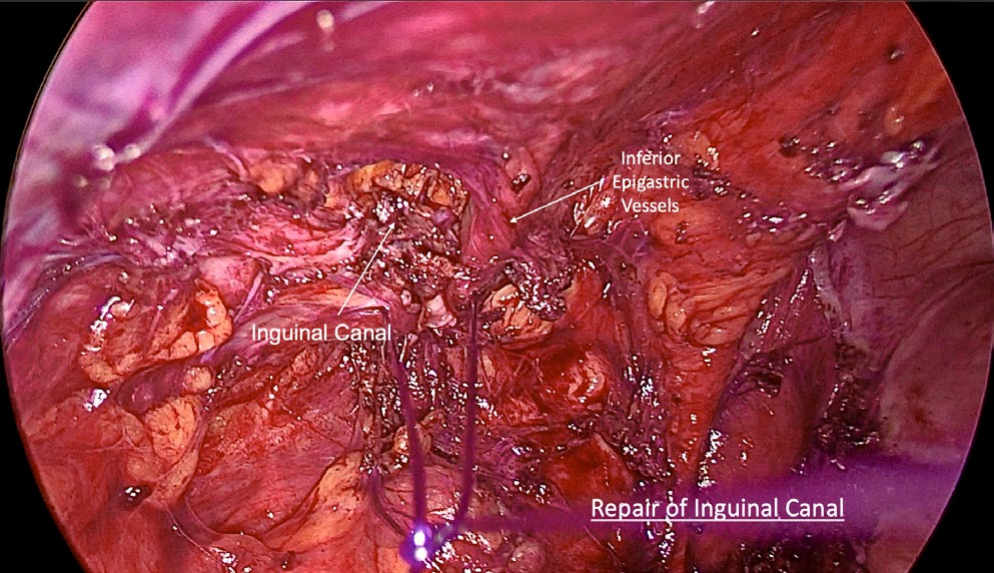
Repair of the left inguinal hernia

## CONFLICT OF INTEREST

The authors declare that they have no conflicts of interest and nothing to disclose.

## AUTHOR CONTRIBUTIONS

KK involved in conception and study design, manuscript preparation, and patient recruitment. NK involved in data collection, statistical analysis, and patient recruitment. MT and DZ involved in data analysis and interpretation, manuscript preparation, and patient recruitment. IC served as responsible surgeon or imager. AP served as responsible surgeon or imager. All authors reviewed the results and approved the final version of the manuscript.

## Data Availability

Data openly available in a public repository that issues datasets with DOIs.
